# Estimating the impact of vaccination: lessons learned in the first phase of the Vaccine Impact Modelling Consortium

**DOI:** 10.12688/gatesopenres.15556.1

**Published:** 2024-09-13

**Authors:** Katy A M Gaythorpe, Xiang Li, Hannah Clapham, Emily Dansereau, Rich Fitzjohn, Wes Hinsley, Daniel Hogan, Mark Jit, Tewodaj Mengistu, T Alex Perkins, Allison Portnoy, Emilia Vynnycky, Kim Woodruff, Neil M Ferguson, Caroline L Trotter

**Affiliations:** 1 Medical Research Council Centre for Global Infectious Disease Analysis, School of Public Health, Imperial College London, London, England, UK; 2 Saw Swee Hock School of Public Health, National University of Singapore, Singapore, Singapore; 3 Bill & Melinda Gates Foundation, Seattle, Washington, USA; 4 GAVI Alliance, Geneva, Geneva, Switzerland; 5 London School of Hygiene & Tropical Health, London, UK; 6 School of Public Health, The University of Hong Kong, Pok Fu Lam, Hong Kong; 7 Department of Biological Sciences, University of Notre Dame, Notre Dame, Indiana, USA; 8 Department of Global Health, University School of Public Health, Boston, Massachusetts, USA; 9 Center for Health Decision Science, Harvard T.H. Chan School of Public Health, Boston, Massachusetts, USA; 10 United Kingdom Health Security Agency, London, UK; 11 University of Cambridge, Cambridge, England, UK

**Keywords:** Vaccine, impact, mathematical modelling

## Abstract

Estimates of the global health impact of immunisation are important for quantifying historical benefits as well as planning future investments and strategy. The Vaccine Impact Modelling Consortium (VIMC) was established in 2016 to provide reliable estimates of the health impact of immunisation.

In this article we examine the consortium in its first five-year phase. We detail how vaccine impact was defined and the methods used to estimate it as well as the technical infrastructure required to underpin robust reproducibility of the outputs. We highlight some of the applications of estimates to date, how these were communicated and what their effect were. Finally, we explore some of the lessons learnt and remaining challenges for estimating the impact of vaccines and forming effective modelling consortia then discuss how this may be addressed in the second phase of VIMC.

Modelled estimates are not a replacement for surveillance; however, they can examine theoretical counterfactuals and highlight data gaps to complement other activities. VIMC has implemented strategies to produce robust, standardised estimates of immunisation impact. But through the first phase of the consortium, critical lessons have been learnt both on the technical infrastructure and the effective engagement with modellers and stakeholders. To be successful, a productive dialogue with estimate consumers, producers and stakeholders needs to be underpinned by a rigorous and transparent analytical framework as well as an approach for building expertise in the short and long term.

## Disclaimer

The views expressed in this article are those of the author(s). Publication in Gates Open Research does not imply endorsement by the Bill & Melinda Gates Foundation.

## 1 Introduction

Health data is rarely available for all geographies and years, yet metrics of health are key to understanding effective interventions, planning for future demand, and ultimately improving population well-being. In addition, some of the metrics of health are themselves not observable; for example, a counterfactual where an intervention was not implemented compared to the reality where it was. Mathematical and statistical modelling has often been used to bridge the gap between incomplete surveillance data and project hypothetical scenarios around interventions, particularly for vaccination. Due to the broad range of uncertainties, differences in data measurement (both geographically and temporally), diverse methodologies, and data gaps, different teams often supply disparate estimates of the same outcome ^1^. This can be beneficial where it raises awareness of data gaps and needs ^2^, as well as the diversity in modelling methodology. However, it can lead to confusion when estimates differ, which can erode trust in the estimation process in general ^3^. Differing estimates of metrics like maternal mortality led to calls for standardised, responsible reporting of global health estimates ^1, 3^. These calls focused on coordinated estimation where stakeholders are engaged from the outset and results are harmonised but not identical; i.e., variation between groups is seen as valuable but consensus can be reached on the main results ^3^. One mechanism for coordinated estimate generation is a consortium of engaged modellers, stakeholders, and policymakers, and this aim motivated the foundation and framing of the Vaccine Impact Modelling Consortium (VIMC).

## 2 Rationale

Reliable estimates of vaccine impact derived from mathematical models are essential to assess the past and future value of vaccine investments, monitor progress towards targets, and guide new vaccine policy decisions. VIMC was established in 2016 to deliver a more sustainable, efficient, and transparent approach to generating disease burden and vaccine impact estimates across the portfolio of vaccines supported by Gavi, the Vaccine Alliance ^1^. VIMC 1.0 created a rigorous methodology and platform for combining and analysing the modelled estimates across twelve vaccine antigens and 112 countries. These estimates had a global, sometimes regional, focus. Such independent and technically sound impact estimates were core to informing the Gavi’s 2021–2025 strategy, and associated replenishment, where the Alliance committed to helping to avert 7–8 million future deaths averted in that time period. There is a continued need for modelling evidence, particularly for informing country, regional, and global vaccination strategies, tracking progress toward Immunization Agenda 2030 (IA2030) goals, and setting new coverage and elimination targets.

In this letter, we review achievements and lessons learnt to date. We also highlight remaining challenges in estimating vaccine impact.

## 3 VIMC structure

**Figure 1.**
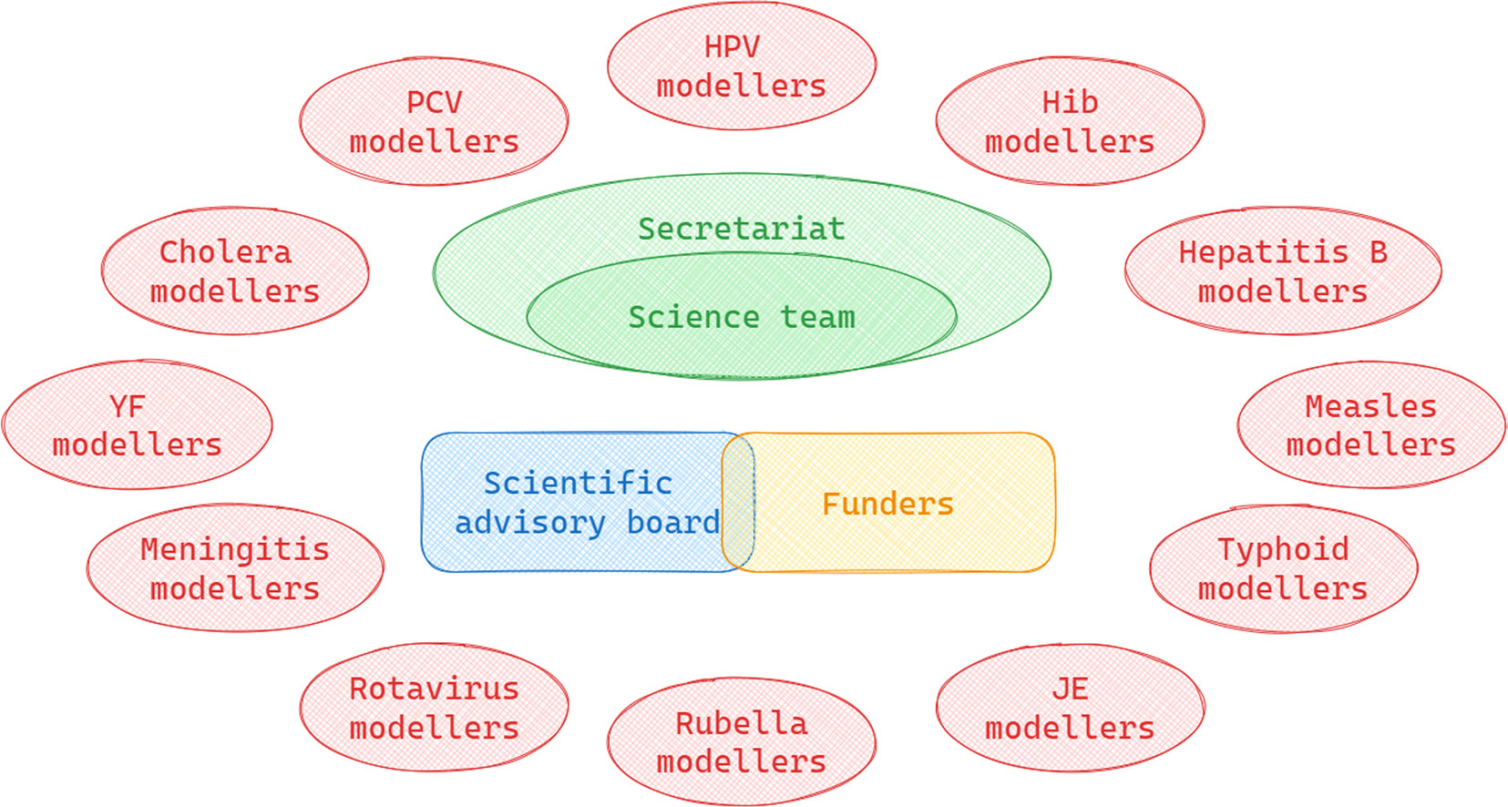
Consortium structure in the first phase of VIMC. HPV=Human Papilloma Virus; Hib=Haemophilus influenzae type B; JE=Japanese Encephalitis; YF=Yellow fever; PCV=Pneumococcal Conjugate Vaccine.

The consortium brought together disease modelling experts who were able to provide vaccine impact estimates for 12 different diseases. Each disease area, shown in red in Figure 1, generally consisted of two disease modelling groups based at different institutions. Many of these groups had been providing impact estimates to Gavi and WHO for several years before the formal establishment of VIMC ^4^. A central secretariat based at Imperial College London incorporated the administrative and science teams, as well as a consortium director. The expert scientific advisory board, which included funder representatives and independent participants, met to discuss the overall scientific priorities of the consortium. They advised on governance procedures, broader strategy and any potential issues relating to the Consortium key outputs the vaccine impact estimates generated in 2017, 2019 and 2021. The funders of the first phase of VIMC were the Bill and Melinda Gates Foundation (BMGF) and Gavi. WHO were also a critical partner as they are the technical agency providing guidance on immunisation.

## 4 Estimating vaccine impact

### 4.1 Defining vaccine impact

The main modelled outcomes for VIMC have been deaths averted, cases averted, and disability-adjusted life years (DALYs) averted. Whilst this does not capture all the benefits of vaccination, such as reduced strain on health systems, costs of illness averted, or economic disruptions due to outbreaks prevented, VIMC’s efforts have supported examination of the wider effect of vaccination through partnership with other research groups ^5, 6^.

One of the roles of VIMC is to define and estimate appropriate metrics to evaluate the benefits of vaccination. Vaccine impact can be valued and estimated in multiple ways, but the methodological approach should be informed by the context and the question of interest. An area of continuing development is the methodology to estimate different values of vaccine impact; e.g., ways to attribute impact either over people’s lifetimes, over a calendar year, or due to one year’s worth of immunisation activities. VIMC has developed a ‘year of vaccination’ approach to valuing impact which considers the longer-term effects of vaccination attributed to the year in which vaccination activities took place ^7, 8^. This approach links more directly with decision making where the outcome of a specific vaccine activity can be examined. The VIMC approach relies on the estimation of ‘impact ratios’: the burden averted per dose, or course of vaccination, over the lifetime of the cohort. The method is not without its limitations, with some of its strengths and drawbacks examined in Echeverria-Londono *et al.* 2021 ^7^; however, it allows for projection of the implications of minor changes in vaccination coverage on impact. This approach, and its efficiency compared to the computational burden of running multiple health impact models, has been invaluable in updating estimates given new data releases, such as the WHO and UNICEF estimates of immunisation coverage (WUENIC), on a short timescale. It is also an approach that has been adopted by Gavi, BMGF, and the IA2030 project team in their estimates ^9^.

### 4.2 Delivery platform

The technical infrastructure required to deliver robust, coordinated, rigorous, and repeatable estimates at scale is considerable. Setting up the VIMC delivery platform infrastructure was more demanding than first anticipated and was a key focus of the early years of VIMC 1.0. VIMC developed an open-source delivery platform that hosts input data, impact estimates, and documentation centrally, with different interfaces for modellers and other stakeholders. The platform allows quality and consistency checks on modellers’ estimates, automated report execution, and includes data visualisation tools.

A robust and reproducible approach to analysis and reports has allowed us to incorporate lessons learnt and methodological improvements throughout the course of VIMC 1.0. For example, through repeatedly running our analyses on data and estimates, we have identified where problems often arise and incorporated diagnostics for issues as they appear. These include anything from automated checks (e.g. are deaths projected to be negative?) to more nuanced verification approaches implemented by the VIMC science team (e.g. are projections between modelling groups notably different?). As a result, each report drafted by the secretariat is not only developed in a collaborative atmosphere focused on best practices such as code review but incorporates learning and diagnostics from the previous six years of research. This is made possible by the robust technical infrastructure, specific software developments like orderly (described below), and support from a diverse range of domain experts including research software engineers. A cross-disciplinary approach that puts reproducibility and reusability as a priority alongside other scientific aims has benefited VIMC efficiency and accountability.

One particular software development that has evolved out of VIMC is orderly ^10^, an open-source R package. Now in its second incarnation, orderly underpins all reports and outputs from the VIMC science team and has been used extensively by the Ebola and COVID-19 Imperial response teams ^11^. orderly versions not only included code but also the data that informed a particular analysis; this is critical when dealing with multiple data inputs and a changing landscape of analytic requests. This way of working is important to address an item on the GATHER checklist for responsible estimate reporting, given that the differences between different iterations of the same analysis can be tracked and fully explained along the entire analysis pipeline ^1^.

### 4.3 Vaccine impact estimates

**Figure 2.**
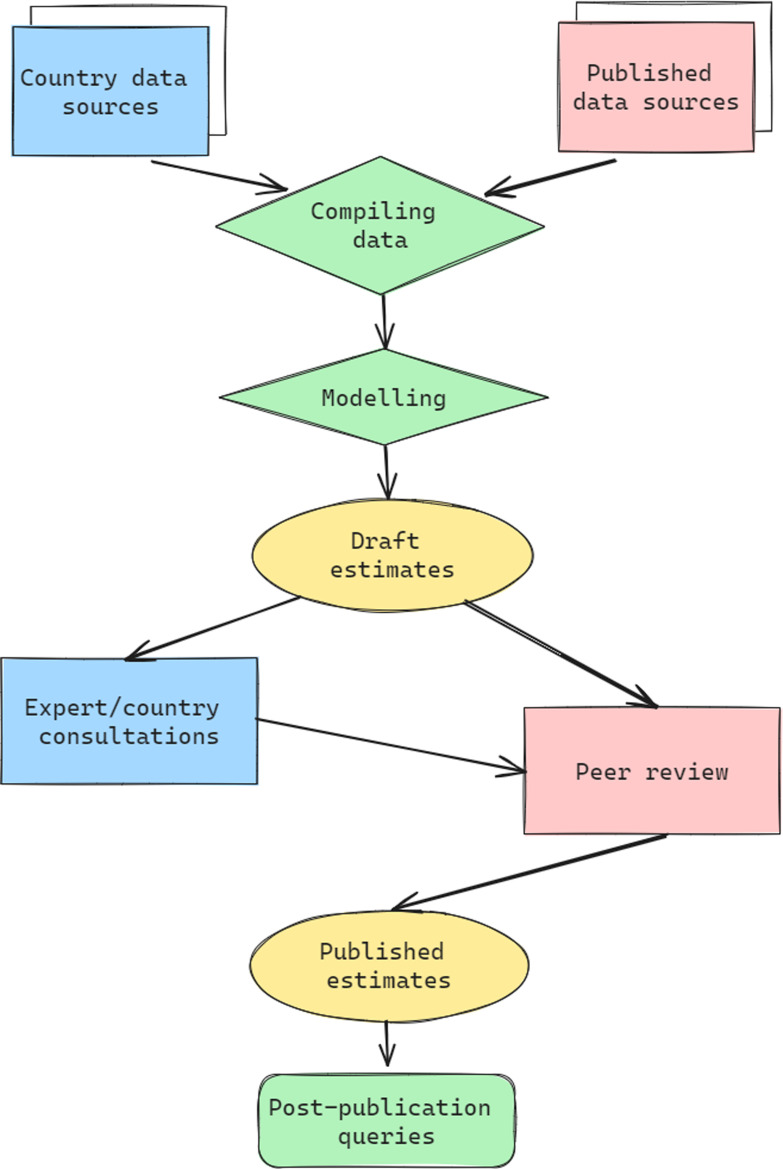
Flow of estimates and input data for the Vaccine Impact Modelling Consortium (VIMC), adapted from 12 to highlight the joining of data flows compared to either the UN agency or academic frameworks.

**Figure 3.**
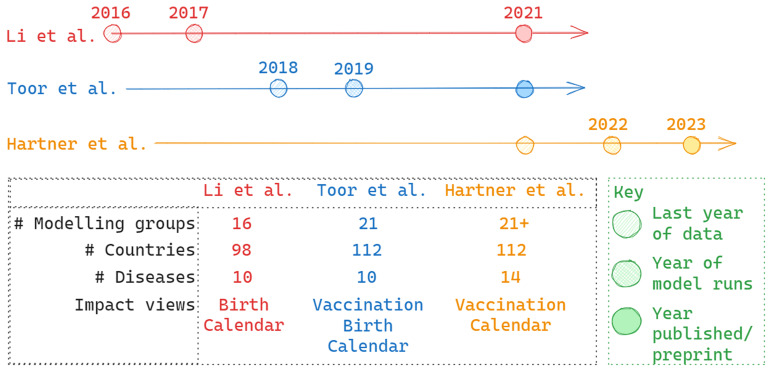
Summary of timing and scope of three consortium wide publications/preprints ^8^, 13, 14. Timelines indicate the last year of included vaccination data, year of model runs and year of publication/ preprint. The table summarises characteristics such as number of included countries or modelling groups included.

Through the framework shown in Figure 2, the VIMC science team collates inputs from country and regional data sources as well as the published literature to supply standardised input data for modellers. These sources include reports on vaccination campaigns, such as described in the WHO weekly epidemiological record, estimates of routine immunisation coverage from WUENIC, or population size estimates from United Nations World Population Prospects. It is critical that inputs such as demography are standardised across modelling groups to ensure comparability of results; this is facilitated by the infrastructure and processes related to the delivery platform. These standardised inputs then inform the modelled estimates of disease burden under each vaccination coverage scenario. The burden estimates from each modelling group are provided to the science team to calculate impact by different views (see Section 4.1) and the results are reported, reviewed, and prepared for publication. Groups were also asked to provide 200 runs of their model for each vaccination scenario to capture parameter uncertainty. This set of ’stochastic runs’ were computationally intensive but vital for communicating the variation in results. This framework has led to some of the key results detailed in Figure 3 and below.

The first consortium-wide publication, presenting results from 16 modelling groups across 98 countries for 10 diseases, estimated that 69 million lives may be saved by vaccination between calendar years 2000 and 2030, 37 million of which were projected to be saved between calendar years 2000 and 2019 ^13^. This paper also presented estimates over the lifetime of individuals vaccinated, projecting a 72% reduction in vaccine-preventable disease (VPD) burden for those born in 2019. These results used available data up to and including 2016 and did not account for the effects of COVID-19 in later years as our analyses pre-dated the pandemic. As such, we may wish to focus on projections for these years of data only: 29.4 million deaths averted between calendar years 2000–2016 and 52.2 million deaths averted for birth years 2000–2016. COVID-19 disruption and the focus on technical development contributed to the time between model runs, in 2017, and publication date in 2021.

The second consortium-wide publication, published in 2021, presented a new vaccine impact view, impact by year of vaccination, and an expanded country scope ^8^. The corresponding estimates were 97 million lives saved by vaccination activities taking place between 2000–2030 with 50 million lives saved by vaccination occurring between 2000–2019. This new vaccine impact view captured the long-term benefits of immunisation, as well as the later burden attributable to HPV or Hepatitis B. The estimates generally overlapped with the first VIMC study, the comparative estimates for 2000–2016 were 30.2 million deaths averted between calendar years 2000 and 2016, and 48.8 million deaths averted for birth years 2000–2016. The time from last year of data and publication date was also reduced, a product of the fully formed technical infrastructure and lessons learnt from the first publication.

The third VIMC paper shown here had a different focus, necessitated by the COVID-19 pandemic ^14^. This work was more motivated by a key policy question at the time: how has the pandemic affected progress in preventing VPDs and the extent to which catch-up vaccination can mitigate losses. There was a much faster progression from data release to modelling to preprint showing the experience in the process and the urgency of the work. Coverage disruptions were found to have led to a 2.7% drop in projected impact for vaccination activities implemented between 2020 and 2030, assuming coverage recovers to pre-pandemic levels in 2025. This paper also examined catch-up activities and projected they could avert 78.9% of excess burden occurring between 2023 and 2030.

## 5 Use cases of model estimates

The following section details some specific case studies, their outputs, and lessons learnt on communicating modelled estimates.

### 5.1 The impact of COVID-19 on vaccine-preventable diseases

We engaged in two major workstreams examining the impact of COVID-19 on vaccination: one in the early months of the pandemic speculatively examining the impact of coverage disruptions, and another after data had become available on what disruptions had occurred ^14, 15^. Both workstreams were developed in collaboration with stakeholders, including WHO and UNICEF, who contributed to defining the scope of the question and communicating the results. The results have informed discussions on prioritisation of vaccination and in understanding the disruption itself ^16^. Furthermore, VIMC estimates were utilised by Abbas *et al.* to make the case for continued routine immunisation during COVID ^17^.

### 5.2 Immunization Agenda 2030

IA2030 is a global vision and strategy set out by the World Health Organisation in 2020 with support from countries and partners to ensure strong immunisation systems, and as part of that, the global immunisation committed to several goals by 2030, including 50m lives saved ^9, 18^. VIMC estimates are a key input into the Immunization Agenda 2030 estimates of vaccine impact. They inform ‘impact goal 1: Prevent disease, indicator 1.1,’ which presents a target number of lives saved through routine vaccination by 2030. VIMC will also be supporting future updates to these indicators. Throughout this process, there has been a productive dialogue between the teams and with the wider vaccine community through the WHO Immunization and Vaccines related Implementation Research Advisory Committee (IVIR-AC). The 50 million deaths averted figure, estimated through the IA2030 project, is based on a ‘year of vaccination’ view of vaccine impact directly informed by VIMC methods. Through the close dialogue between VIMC and the IA2030 project team, and the adoption of VIMC methods by the IA2030 project team, a wider level of feedback and scrutiny has been sought, which enhances both the IA2030 and VIMC estimate generation.

### 5.3 Uses of estimates by BMGF and Gavi

Gavi has utilised VIMC estimates to develop health impact targets in their 5.0 investment opportunity, report on progress in annual reports, and compare impact and value for money of new vaccines being assessed as part of the Gavi Vaccine Investment Strategy to that of current portfolio vaccines ^18– 22^. Gavi uses (a) the impact by year of vaccination method to reflect the full value /long-term impact of vaccination as well as to place vaccines that impact mortality later in life (e.g. HepB and HPV), on the same playing field as vaccines that have more immediate impact such as measles. (b) the average impact across multiple disease models for each antigen in order to account for uncertainty in estimated impact due to model differences. Both Gavi and IA2030 are using the same approach for key reporting on Gavi 5.0 and IA2030 estimates to ensure clarity and alignment. BMGF has used estimates for internal strategy development and reporting.

## 6 Lessons learnt and remaining challenges

To produce robust, reproducible estimates at scale, the underlying technical infrastructure is critical and requires development in its own right. In the first phase, this required more effort than initially envisaged, but the consortium is now reaping the rewards of a solid technical foundation. Other modelling consortia/ projects may need to consider the technical needs alongside research plans to achieve results efficiently, though this will be specified by the consortia objectives.

Mathematical modelling of infectious diseases is often limited by data availability. This is the case across the diseases considered in the consortium, where even the least neglected infections still have a wish-list for further data to inform the model projections. Furthermore, there may be disparities between complementary data sources, or uneven access to data sources due to data sharing agreements. Even in the face of improved surveillance, there will always be data limitations and so the focus should be on transparently reporting uncertainty and underlying assumptions in model estimates, identifying key data gaps, as well as effectively synthesising available data. VIMC places an emphasis on the ability to report on appropriate descriptions of uncertainty in its model criteria, which encourages such practices among its models. Modellers are also well-placed to communicate these uncertainties and aid in the interpretation of the results.

The timeline in Figure 3 highlights the delay between the last year of collected data and publishing the resulting estimates of vaccine impact. This delay has decreased over the course of the first phase of the consortium, but there will continue to be a lag between data and results. The policy question will define how long an ‘acceptable’ delay is, but timely reporting of estimates should be a focus of global health modelling and (relatively) new ways of working, such as using preprint servers, producing shorter reports and blog posts, and communicating through other routes are needed to disseminate results effectively (with the appropriate caveats around peer-review). Organisational approval processes also need to consider the immediacy of results.

There is a trade-off between model and data developments and consistent messaging. Through the course of the first phase of VIMC, we produced multiple estimates of vaccine impact and needed to thoroughly explain changes in models, data inputs, future coverage assumptions, and scope. This is essential to ensure confidence in the results and is partly facilitated by transparent reporting of uncertainty and underlying assumptions. VIMC also implemented a peer-peer review process as models were developed. The routine reporting of uncertainty in estimates has been well utilised in climate science such as the IPCC reports but is less prevalent in reporting epidemiological quantities ^23^. This is a focus of extensive work in terms of model estimation but the translation of uncertainty into effective communication is an ongoing challenge.

It is well accepted, although less widely implemented, that stakeholders should be engaged from the outset to help define the question, as well as review and disseminate the output ^24, 25^. Decision-makers in the countries that bear the greatest burden of disease are also key stakeholders, whose input into key modelling priorities is essential. However, stakeholder bandwidth can be a challenge, as the same individuals are often invited to contribute to multiple projects. Developing frameworks so that the right individuals and institutions can effectively contribute to question development as well as other stages of the modelling process is critical and an area of active work. Similarly, the dialogue should be two-way for modelling groups to effectively engage with framing and operationalising the question and priorities at hand.

The geographic scope of the modelling in the first phase of VIMC was not reflected in the distribution of modelling groups who were disproportionately based in HICs. This may have affected the types of analyses undertaken and the presentation of the outputs, as well as missing valuable insights from experts in LMICs ^26^. As with involving stakeholders from the start of the of the modelling, we have learnt that it is important for VIMC to be more inclusive of modellers from LMICs and be mindful of who conducts the analysis from the outset. This is both a question of linking institutions and individuals to ensure a productive and inclusive environment as well as promoting opportunities to build expertise and reciprocal relationships.

## 7 The next phase of VIMC

Having established the technical infrastructure and supported model development in VIMC 1.0, in its next phase beginning 2022, VIMC is expanding its work to address specific policy-relevant questions, as well as produce large-scale estimates of vaccine impact. Furthermore, VIMC 2.0 is building a more diverse, engaged network of modellers inclusive of those based in low-and-middle-income countries (LMICs) through training, fellowships, and collaborative networks.

### 7.1 Vision by 2027

By the end of the five-year grant in 2027, VIMC expects to be:

- A standardised source providing reliable and accessible estimates of vaccine impact across the Gavi portfolio.
- Able to address critical modelling-related vaccine policy questions raised by international stakeholders who will be dynamically engaged in our work.
- Able to demonstrate translation from VIMC modelling to real-world policy that improves health outcomes.
- A diverse international community of vaccine impact modellers, inclusive of modellers in LMICs, that clearly articulates its own mission and adds value as a partner to other vaccine modelling groups and entities.
- A significant global provider of training in infectious disease modelling and its application to vaccine preventable diseases for both modellers and policymakers using modelling to inform decision making.

### 7.2 Strategy

**Figure 4.**
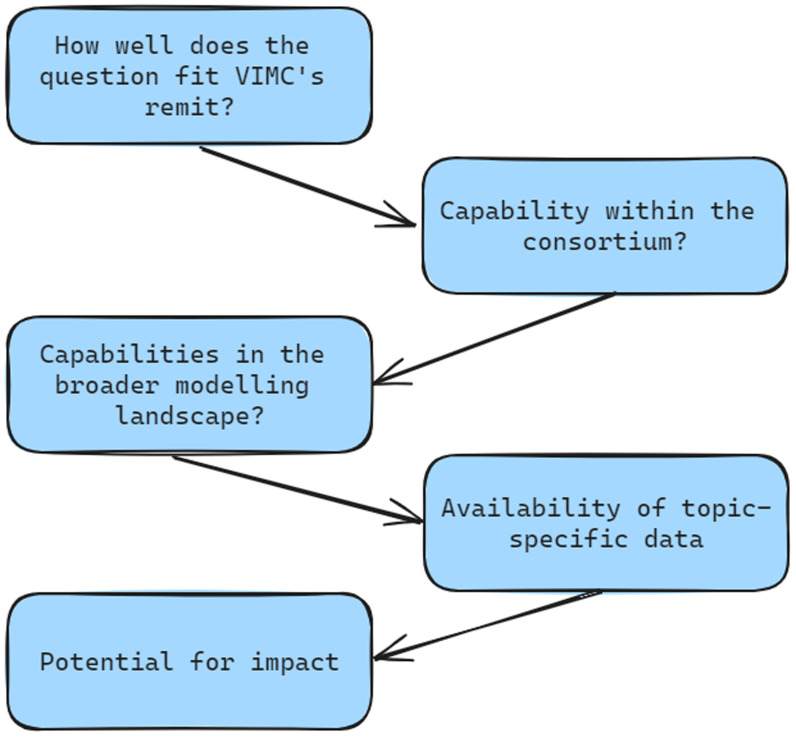
Considerations for prioritisation of modelling and policy questions.

A major update of estimated vaccine impact is planned for 2023/4 to support Gavi 6.0 and IA2030, with annual updates to support the ongoing work of Gavi, BMGF, WHO, and other stakeholders. Prior methodological and technical investments in VIMC 1.0 deliver the infrastructure which allows standardised model outputs to be submitted, rigorously checked, and analysed across a broad range of vaccines. Fewer major updates will allow space for a more proactive and responsive research programme providing evidence for vaccine policy. Modelling groups with foundational support will be commissioned to respond to specific policy questions in their disease area. Further requests for proposals (RfPs) will be issued by the secretariat for vaccine impact modelling that falls outside the scope of the consortium’s existing model capability. For all RfPs (including those for new foundational models), we will particularly encourage applications from modelling groups in countries with a high disease burden. To ensure that our research is timely, relevant, and useful, we will strengthen our relationships with key stakeholders and policymakers and an active process of soliciting research questions will be led by the VIMC Director. These questions will be prioritised in line with VIMC resources and according to a systematic process, see Figure 4 for considerations for prioritisation.

VIMC 2.0 is working with modelling groups in Africa and Asia who are supported by parallel initiatives. Additionally, we will invite individuals from across our network to become affiliate members of VIMC. Affiliate membership will facilitate access to VIMC resources, including regular webinars, training opportunities, and a buddy scheme for PhD students to support peer-to-peer learning and encourage the development of international links between students. Training activities will be co-created in partnership with LMIC modellers and a bespoke vaccine modelling course will run in alternate years complemented by visiting fellowships of longer duration.

One of the key strengths of VIMC is research capacity across multiple pathogens and antigens, enabling the consortium to examine cross-cutting issues in VPDs. Initial specific research questions will focus on optimising the use of current vaccines by examining the potential for improvement in schedules, campaign synergies, and efficiency in the use of stockpiles. Furthermore, inequity in vaccine access due to socioeconomic or geographic factors, and the differential benefits of vaccination in individuals with multiple deprivations, are key areas of research where VIMC can build on its existing expertise.

## 8 Conclusions

Consistent and clear estimates of intervention impact are vital for future planning. The Vaccine Impact Modelling Consortium has changed the landscape of how vaccine impact estimates are generated and reported, ensuring that robustness and reproducibility are integrated at each step. While modelled estimates can to some extent complement and support attempts to address missing data, evidence from high quality surveillance remains essential. To be successful, a robust and productive dialogue is needed between modellers, stakeholders, and estimate consumers to enhance understanding and trust. This should be underpinned by a rigorous and transparent approach to generating estimates, as well as a multi-layered approach to building expertise on both the short and long term.

## Ethics and consent

Ethical approval and consent were not required.

## Data Availability

No data are associated with this article. Figures were drawn using Excalidraw https://excalidraw.com/.

## Footnotes

^1^ a public private partnership that aims to increase the use of new and under used vaccines
