# Supplementary material for: Estimating the impact of vaccination: lessons learned in the first phase of the Vaccine Impact Modelling Consortium
Source: Gates Open Res. 2024 Sep 13;8:97. [Version 1] doi: 10.12688/gatesopenres.15556.1 (PMC11467163; doi:10.12688/gatesopenres.15556.1)
Supplement: Peer review history

# Reviewer report: Reviewer response for version 1

**DOI:** 10.21956/gatesopenres.16912.r38051

**Published:** 2024-10-11

**Authors:** David Regan

**Affiliations:** 1 The Kirby Institute, University of New South Wales,, Sydney, Australia

This Open Letter reports on progress, achievements, challenges and lessons learnt in the first five years since establishment of the Vaccine Impact Modelling Consortium (VIMC 1.0). The Consortium was established to provide estimates of impact of GAVI-supported vaccination programs to inform planning for future demands that are robust, reproducible, and in which policy makers and end users can have confidence. This is an important and ground-breaking undertaking that should be of interest to readers and, in particular, to those engaged in modelling for public health.

 The Letter provides a fairly comprehensive overview of the rationale, approach, and lessons learnt, as well as some use-case examples. The report does not provide a complete compendium of output from the Consortium, and this is perhaps outside the scope of the Letter. There are, however, some aspects of this undertaking for which I think more detail could be provided.

 While I realise that the primary function of the Consortium is to evaluate the impact of vaccination programs that are already being implemented, I think it is worth mentioning the very important role of modelling for informing implementation strategy prior to roll-out, particularly in regard to decisions around funding and target populations.

 I also realise that it is beyond the scope of the Letter to provide a detailed description of the modelling methodology and paradigms used, but I do think a summary of these would be of interest to many readers along with additional references and links to any open-source code. The authors could also consider a technical appendix if this is appropriate with the Letter format.

 In a similar vein, the authors discuss the importance of the technical infrastructure required to deliver robust and reproducible estimates, but other than reference to the *orderly* software package, there is little detail of what these requirements are or what they refer to. The Letter would benefit from some examples of what the technical needs are (software, hardware, technical expertise, etc.) and how these needs have been met.

 On the subject of examples, the authors state that the Consortium’s approach relies on the estimation of “impact ratios”, noting that this approach has some limitations but providing no examples, preferring just to provide a reference. This is a rather key element of the methodology, and I think a brief discussion of strengths and weaknesses is warranted.

 Finally, In the Discussion/Conclusion section of the Letter, I feel the authors could provide more of a big picture view of how such undertakings can inform public health policy around vaccination and other interventions to improve health, beyond the Gavi supported programs, and where such efforts could be focused.

Does the article adequately reference differing views and opinions?

Yes

Are all factual statements correct, and are statements and arguments made adequately supported by citations?

Yes

Is the Open Letter written in accessible language?

Yes

Where applicable, are recommendations and next steps explained clearly for others to follow?

Yes

Is the rationale for the Open Letter provided in sufficient detail?

Yes

Reviewer Expertise:

infectious disease epidemiology; mathematical modelling; vaccination; sexually transmissible infections

I confirm that I have read this submission and believe that I have an appropriate level of expertise to confirm that it is of an acceptable scientific standard, however I have significant reservations, as outlined above.

# Reviewer report: Reviewer response for version 1

**DOI:** 10.21956/gatesopenres.16912.r38053

**Published:** 2024-10-10

**Authors:** Gilberto Gonzalez-Parra

**Affiliations:** 1 Department of Mathematics, New Mexico Tech, Socorro, USA

In this article a framework and summary about a project to estimate the global health impact of immunisation is presented. The authors used several methods to quantify the impact of immunization. The topic is very challenging due to the many effects that immunization has on the population and on the public health. Moreover, some of these effects are difficult to quantify and observe.  In addition, the authors deal with the communication part of spreading the benefits of vaccination programs. At the end the authors explain main aspects that can be concluded related to the estimation of the effects of vaccination on the population and the Vaccine Impact Modelling Consortium (VIMC).

 The authors proposed the deaths averted, cases averted and DALYs as the output metrics to measure the benefits of vaccination. As the authors mentioned there are other benefits. One main aspect of the proposed methodology to measure benefits is to use the ratio of averted and dose. The authors developed codes in open-source R package.  The VIMC was divided in groups depending on the diseases and groups provided 200 stochastic runs of their model for each vaccination scenario to capture parameter uncertainty.

 Another aspect that VIMC project tackle was the estimation over the lifetime of individuals vaccinated, projecting a 72% reduction in vaccine preventable disease (VPD) burden for those born in 2019.

 The phrase “To be successful, a productive dialogue with estimate consumers, producers…” that appears in the abstract is confusing for the readers. It is not clear in the context of estimating effects of vaccination.

 In page 2 the authors wrote in this letter but in the abstract in this article. It is a little confusing since in general a letter is more like an opinion on some subject.

 Gavi and BMGF should be defined. Many readers would not know for what these stands.

 In summary the aims of VIMC are excellent. In this article some general conclusions are presented. The article does not deal with the specific methodologies that were used to obtain the estimates of the benefits of immunization which is a main goal of the VIMC. The article presents some lessons that should be mentioned for future works. For instance, VIMC describes or considers several aspects related to uncertainty related to the data, modeling, etc.  The general results presented in this article are related to a first phase of the VIMC.  Nevertheless, the article mentions general future directions of the next phase which seems reasonable from a scientific perspective. Validation of the results was not addressed specifically, but I think that is part of other articles. The authors can clarify this point more explicitly.

 The authors concluded that robust estimates are important for future planning. The VIMC also considers the reproducibility of the results, and its results complement other relevant data related to the benefits of vaccination.

Does the article adequately reference differing views and opinions?

Yes

Are all factual statements correct, and are statements and arguments made adequately supported by citations?

Yes

Is the Open Letter written in accessible language?

Yes

Where applicable, are recommendations and next steps explained clearly for others to follow?

Yes

Is the rationale for the Open Letter provided in sufficient detail?

Yes

Reviewer Expertise:

Mathematical modeling of infectious diseases, mathematical analysis, numerical analysis, optimization.

I confirm that I have read this submission and believe that I have an appropriate level of expertise to confirm that it is of an acceptable scientific standard.

# Reviewer report: Reviewer response for version 1

**DOI:** 10.21956/gatesopenres.16912.r38050

**Published:** 2024-09-24

**Authors:** Zakaria Yaagoub

**Affiliations:** 1 Laboratory of Mathematics, Computer Science and Applications, Faculty of Sciences and Technologies, University Hassan II of Casablanca, Mohammedia, Morocco

This paper examines the first five years of the Vaccine Impact Modeling Consortium, describing the methods used for estimation, the technical infrastructure required for reproducibility, and the definition of vaccine impact. It draws attention to the challenges encountered, lessons learned, and applications and impacts of these estimates. The significance of model estimates in identifying theoretical scenarios and data gaps to support surveillance is also covered in the article. In order to create reliable and consistent estimates, the Vaccine Impact Modeling Consortium has created methods. It also highlights the importance of an open analytical framework and proactive stakeholder engagement in order to develop future expertise. In my opinion, the paper presents interesting insights and merits indexing after addressing the following minor changes:

 1. Add more comments regarding all figures included in the paper especially Figure 4.

 2. Write about mathematical modeling especially models that integrate vaccination strategy and talk about the effect of this strategy to eradicate the infectious diseases. You can consider these works :[1],[2],[3]

 3. Improve the conclusion. Give some future direction.

Does the article adequately reference differing views and opinions?

Yes

Are all factual statements correct, and are statements and arguments made adequately supported by citations?

Yes

Is the Open Letter written in accessible language?

Yes

Where applicable, are recommendations and next steps explained clearly for others to follow?

Yes

Is the rationale for the Open Letter provided in sufficient detail?

Yes

Reviewer Expertise:

Modeling Mathematics; Biomathematics; System dynamics; Fractional calculus; Infectious diseases.

I confirm that I have read this submission and believe that I have an appropriate level of expertise to confirm that it is of an acceptable scientific standard.

## References

[1] : Global Stability of Multi-Strain SEIR Epidemic Model with Vaccination Strategy. *Mathematical and Computational Applications* .2023;28(1) : 10.3390/mca28010009 10.3390/mca28010009

[2] : On a two-strain epidemic mathematical model with vaccination. *Comput Methods Biomech Biomed Engin* .2024;27(5) : 10.1080/10255842.2023.2197542 632-650 10.1080/10255842.2023.2197542 37018044

[3] : Computational dynamics of a fractional order model of chickenpox spread in Phuket province. *Biomedical Signal Processing and Control* .2024;91: 10.1016/j.bspc.2024.105994 10.1016/j.bspc.2024.105994
